# The cross- ethnic variations in the prevalence of headache and other somatic complaints among adolescents in Northern Israel

**DOI:** 10.1186/1129-2377-14-21

**Published:** 2013-03-04

**Authors:** Jacob Genizi, Isaac Srugo, Nogah C Kerem

**Affiliations:** 1 Pediatric Neurology Unit, Bnai Zion Medical Center, Haifa, Israel; 2 Pediatric Department, Bnai Zion Medical Center, Haifa, Israel; 3 Adolescent Medicine Unit,Bnai- Zion Medical Center, Bruce Rappaport Faculty of Medicine, Technion, Haifa, Israel

**Keywords:** Headache, Somatic complains, Adolescents, Ethnicity

## Abstract

**Background:**

Headache is the most common cause for chronic or recurrent pain in childhood and adolescence. Chronic pain may have a long-term effect on adolescents. It might contribute to functional limitations, such as poor school attendance, and it may adversely affect development of healthy social relationships. The aim of our study was to examine the cross- ethnic variation in the prevalence of headache in a non- clinical sample of adolescents in Northern Israel and to learn about its association to other somatic complaints.

**Methods:**

A self-administered, anonymous questionnaire was presented to 2,088 tenth grade students attending 19 high-schools in Northern Israel (all the public high schools within two districts). Participants were Jews and Arabs, the latter including Muslim, Christians, and Druze, aged 15 to 16. Parental and student consent was obtained from all participants. The study was approved by the IRB of our institution.

**Results:**

All 2088 questionnaires were returned although only 2019 were usable and analyzed. Arab adolescents comprised 55% (1117) of the analyzed sample and Jews 45% (902), 56% of participants were girls. Of the Arab participants, 18.6% reported having frequent headaches (girls 25.3%, boys 9.1%, P<0.0001) much less than their Jewish peers (P<0.0001) among whom 27.9% reported having frequent headaches (girls 35.6%, boys 19% P<0.0001). Other somatic complaints such as abdominal pain, palpitations, disordered sleep and fatigue were more frequent in adolescents (Jews and Arabs, girls and boys) who suffered from headaches than in their peers who did not report having headaches (P<0.0001), the same pattern observed in the Jewish and the Arab group.

**Conclusions:**

Headache is a frequent complaint among adolescents in Northern Israel. Jewish adolescents reported having headaches more frequently than their Arab peers. Those who suffered from frequent headaches also reported having significantly more other somatic complaints than adolescents without headaches. Girls had more somatic complaints then boys in the two ethnic groups.

## Background

Headache, abdominal pain, and fatigue are frequently reported somatic complaints among adolescents [1]. Studies worldwide show that girls, compared to boys, report higher prevalence of these symptoms [2,3] and are at greater risk for experiencing these symptoms concurrently [4,5]. The experience of suffering from chronic pain may have long-term effects on the development of adolescents and may contribute to functional limitations, that would adversely affect school attendance, development of healthy social relationships, and participation in a variety of youth activities [6,7]. Headache is the most common cause for chronic or recurrent pain in childhood and adolescence [8]. Geographical differences in headache prevalence have been demonstrated in migraine, showing higher prevalence in Europe and the Middle East compared to the USA and the Far East [9]. Demographic influence was described worldwide as well, showing higher headache prevalence among urban, compared to rural population [10,11]. Ethnic differences in headache prevalence were demonstrated in Singapore between Malay and non-Malay ethnicity [12], in Brazil between Caucasians and other ethnicities [13] and in the United States between American Indians, Caucasians and Afro Americans [14]. Jews and Arabs are the two major ethnic groups that comprise the Israeli society. The language used for teaching in all public schools in Israel is either Hebrew or Arabic, and some of the subjects taught, such as: history, religion, literature etc. differ according to the declared ethnicity of the school. In cities where the population is mixed - students can chose what school to attend, yet most families chose to send their children to schools teaching in their mother-tongue and focusing on history and culture of their religion. Many cities / villages in Israel are mostly "single ethnic" so students attend either Arabic or Hebrew speaking schools. There is a slowly growing number of schools teaching in both languages, and/or in other languages (mostly English), but most of them are privately managed and were not included in this study.

In this study, we were interested in whether there were cross-ethnic differences in the prevalence of headache and its association with other somatic complaints among Israeli adolescents.

### Methods

Patients –10th grade students (between the ages of 15–16) from all the public high-schools within two districts in Northern Israel (19 high schools overall), attending Jewish and Arab schools. Out of about 2250 students enlisted to the 10th grade in the 19 schools surveyed, 2088 questionnaires were filled, and 2019 (97%) had all the demographic parameters and were analyzed. Fifty five percent (1117) of the students were of Arab descent, and forty five percent (902) were Jewish. Parental consent and students' assent was obtained, as well as an IRB approval.

Methods - A self-administered, anonymous, pen and paper questionnaire was presented to tenth grade students in their classrooms during 2006–2007. Research purpose, contact numbers of adolescent medicine clinics in the area, and issues of confidentiality were discussed with the students prior to the procedure. The questionnaire was originally written in Hebrew, and then translated linguistically and culturally by two independent translators to Arabic, using back translation and pretest, according to recommendations for linguistic and cultural translation [15].The language of the questionnaire handed out was according to the students' mother tongue. The questionnaire included questions about the student's ethnicity (Jewish or Arabic), and within the latter ethnic group asked about their religion (Islam, Christianity, and Druze), there were questions about demographics, known medical diagnoses and prevalence of somatic complaints including headache, abdominal pain, fatigue, dizziness, disordered sleep, etc. Questions regarding somatic complaints related to the year prior to questionnaire completion. Frequency of complaints was defined as every day, every week, seldom or never. The questionnaire did not include question characterizing different headache types (i.e. Migraine, Tension).

Statistical analysis- Bivariate analyses of the associations between headache frequency, gender, ethnicity, and other somatic complains were conducted with Mann–Whitney U tests. Multivariate analyses of the associations between headache frequency, gender, ethnicity and other somatic complains, were conducted with nominal regressions.

## Results

Out of 2019 questionnaires eligible for the study, fifty five percent (1117) of the students were of Arab descent, and forty five percent (902) were Jewish. There were significantly more girls in the Arab group compared to the Jewish group (p=.037). See Table 1 for demographic characteristics of the students surveyed. The prevalence of frequent headaches (at least one episode of headache per week during the past year) was higher in the Jewish than in the Arab group of students (p=0.001). Of the Arab students 18.6% (208) reported having frequent headaches, of whom 15.1% (169) suffered headaches on a weekly basis and 3.5% (39) reported having headaches daily. 27.9% of the Jewish students reported having frequent headaches, 24.2% (216) weekly and 3.7% (33) on a daily basis. Girls reported suffering from headaches more frequently than boys, and the differences between genders remained significant within each ethnic group (p<0.0001). See Table 2 for the prevalence of frequent headaches within each ethnic group.

**Table 1:** Demographic information

	**Boys**	**Girls**	**Total**
	**N**	**N**	**N**
	**(%)**	**(%)**	**(%)**
**Jews**	414 (45.9)	488 (54.1)	902 (44.7)
**Arabs**	461 (41.3)	656 (58.7)	1117 (55.3)
**Total**	875 (43.3)	1144 (56.7)	2019 (100.0)

**Table 2:** The prevalence of frequent headaches in the Arab and Jewish groups

**Ethnicity**	**Boys**	**Girls**	**Total**	**Gender differences (p)**
**Arab students**	42/461	166/656	208/1117	**(p<.0001)**
	(9.1%)	(25.3%)	(18.6%)	
**Jewish students**	78/414	171/488	249/902	**(p<.0001)**
	(19.0%)	(35.6%)	(27.9%)	
**Ethnic Difference (p)**	P=0.002	P=0.008	P=0.0001	

The prevalence of other somatic complaints that students were asked about, such as, abdominal pain, fatigue, palpitations, dizziness, and disordered sleep, also showed the same trends: Jewish students overall reported of more frequent complaints than their Arab peers in all but disordered sleep complaints (p<0.001), See Table 3 for differences between the ethnicities with regards to prevalence of somatic complaints. Within both ethnic groups complaints about abdominal pain, fatigue, palpitations and dizziness were reported more frequently by girls than by boys*.* See Tables 4 and 5 for the prevalence of somatic complaints for the Arab and the Jewish groups. The order of prevalence of the different somatic complaints differed between the two ethnic groups: The most abundant complaints for the Arab students were headaches and fatigue, then disordered sleep and abdominal pain (in a declining order). For the Jewish students the most common complaint was fatigue (over 50% of the Jewish girls reported feeling frequently fatigued!), then headaches, abdominal pain and dizziness.

**Table 3:** Comparison of Arab and Jewish students' reports of other somatic complains

**J=Jews**	**Total sample**		**Boys**		**Girls**	
**A=Arabs**	**(N=2019)**		**(N=875)**		**(N=1144)**	
**Complaint**	Z		Z		Z	
	(p)		(p)		(p)	
**Abdominal pain**	**11.15**	J > A	**9.24**	J > A	**8.22**	J > A
	**(p<0.001)**		**(p<0.001)**		**(p<0.001)**	
**Palpitations**	**5.11**	J > A	1.25		**5.94**	J > A
	**(p<0.001)**		(p=0.213)		**(p<0.001)**	
**Fatigue**	**15.81**	J > A	**10.64**	J > A	**12.47**	J > A
	**(p<0.001)**		**(p<0.001)**		**(p<0.001)**	
**Dizziness**	**6.21**	J > A	**4.51**	A > J	**5.05**	J > A
	**(p<0.001)**		**(p<0.001)**		**(p<0.001)**	
**Disordered Sleep**	2.17		2.40		1.20	
	(p=0.030)		(p=0.017)		(p=0.229)	

**Table 4:** The prevalence of somatic complaints and the differences between genders within the Arab group of students

**Complaint**	**Boys**	**Girls**	**Total**	**Gender Difference**
	**(N=461)**	**(N=656)**	**(N=1117)**	**Z (p)**
**Headaches**	42	166	208	**(p<.0001)**
	(9.1%)	(25.3%)	(18.6%)	
**Fatigue**	59	144	203	**3.90**
	(12.8%)	(22.0%)	(18.2%)	**(p<0.0001)**
**Disordered Sleep**	43	116	159	**3.93**
	(9.3%)	(17.7%)	(14.2%)	**(p<0.0001)**
**Abdominal pain**	30	107	137	**4.92**
	(6.5%)	(16.3%)	(12.3%)	**(p<0.0001)**
**Palpitations**	54	65	119	0.96
	(11.7%)	(9.9%)	(10.7%)	(p=0.34)
**Dizziness**	88	31	119	**3.57**
	(13.4%)	(6.7%)	(10.7%)	**(p<0.001)**

**Table 5:** The prevalence of somatic complaints and the differences between genders within the Jewish group of students

**Complaint**	**Boys**	**Girls**	**Total**	**Gender Difference**
	**(N=414)**	**(N=488)**		**Z (p)**
**Fatigue**	144	246	390	**4.83**
	(35.0%)	(51.0%)	(43.6%)	**(p<0.0001)**
**Headaches**	78	171	249	**5.48**
	(19.0%)	(35.6%)	(27.9%)	**(p<o.0001)**
**Abdominal pain**	38	145	183	**7.65**
	(9.3%)	(30.1%)	(20.5%)	**(p<0.0001)**
**Dizziness**	30	87	117	**4.77**
	(7.4%)	(18.4%)	(13.3%)	**(p<0.0001)**
**Palpitations**	31	72	103	**3.50**
	(7.6%)	(15.2%)	(11.7%)	**(p<0.001)**
**Disordered Sleep**	37	64	101	**2.01**
	(9.0%)	(13.3%)	(11.3%)	**(p=0.05)**

The prevalence of all somatic complaints was significantly higher among students who complaint about frequent headaches (once a week) as compared to those with non- frequent headaches (once a month). It was also higher in the group having non frequent headaches when compared to those with no headaches at all. See Table 6 for prevalence of somatic complaints in the different headache frequencies groups. The more frequent somatic complaints of students from both ethnicities who reported having headaches were fatigue, abdominal pain, and disordered sleep, then dizziness and palpitations.

**Table 6:** The prevalence of somatic complaints by the frequency of headache

**Complaint**	**No headaches**	**Non frequent headaches**	**Frequent headaches**	**Difference**
	**(N=385)**	**(N=1166)**	**(N=457)**	**χ**^**2 **^**(2) (p)**
**Fatigue**	31	313	246	**220.09**
	(8.1%)	(26.9%)	(53.9%)	**(p<0.0001)**
**Abdominal pain**	10	162	146	**143.49**
	(2.6%)	(13.9%)	(32.1%)	**(p<0.0001)**
**Dizziness**	6	78	152	**275.16**
	(1.6%)	(6.7%)	(33.8%)	**(p<0.001)**
**Disordered Sleep**	22	130	108	**67.52**
	(5.7%)	(11.2%)	(23.7%)	**(p<0.0001)**
**Palpitations**	15	107	98	**76.30**
	(3.9%)	(9.2%)	(21.7%)	**(p<0.0001)**

## Discussion

Northern Israel is populated heterogeneously by Jews and Arabs, yet most schools are attended by students from the same ethnicity. As clinicians who see patients from both ethnicities we were interested in investigating whether there were cross-ethnic differences in the prevalence of headaches and other somatic complaints, and cross-ethnic differences in regards to gender and somatic complaints.

Our study showed, that the prevalence of frequent headaches was significantly different between the two ethnic groups: It was much higher in Jewish adolescents than in Arab adolescents (27.9% Vs 18.6%, respectively, p=0.001). The differences between boys and girls within each ethnic group were significant, with girls reporting more frequent headaches than boys (p<0.0001).

The reported prevalence of headaches in children and adolescents in the literature ranges from 19.5% to 93.3% [16]. The wide range of prevalence depends on many factors, mostly on methodological factors, but geographical, sociocultural and ethnic factors have been suggested as well. Rhee [14] reported that 30% of the adolescents in the United States complained about having recurrent headaches occurring at least once a week. American Indians experienced the highest rate of recurrent headaches (35.6%) followed by Caucasians adolescents (32.1%). Two older studies that were conducted in the United States have also looked at ethnic differences, mostly between Caucasians and Afro-Americans, in an adult samples [17,18], and demonstrated a higher prevalence of headaches among Caucasians.

The prevalence of headaches in the Arab ethnicity was studied in few countries in the Middle East: Al Jumah et al. [19] reported high prevalence of recurrent headaches, either Migraine (boys 8.6% girls 10.3%) or non- migraine (boys 49.9% girls 53%), among Saudi Arabia adolescents. These rates are much higher than in our study. Isik et al. [20] reported headache prevalence in Turkish adolescents to be 31.4% with low Migraine prevalence (3.3%), quite similar to headaches prevalence in the Jewish population in our study, and found no differences between males and females, very different than in our study.

The difference in headache prevalence between Arab and Jewish adolescents came as a surprise for the investigators, as lower socioeconomic status is considered to be a risk factor for headaches in adolescents [7], and in average the socioeconomic status of the Jewish population in Israel is higher than that of the Arab population. Being concerned about unemployment has also been reported as a major stress factor for adolescents in Western Europe, that can aggravate headaches, yet unemployment is more prevalent among Arabs than Jews in Israel.

The ethnic differences might be explained by various reasons; all are speculative and need further evaluation: Is it because more pressure is put, as a generalization, on the Jewish adolescents to be high achievers in their academic work? About 60% of the Jewish high school students pass the final exams in comparison to 41% of the Arab students (26.5% of the Jewish population complete academic degrees in comparison to 10.6% of the Arab population [21]. Could it be explained by relatively high stress levels Jewish adolescents in high school experience due to concerns about their future army service? (All Jewish citizens are expected to serve in the IDF after graduating from high school) Could it be a higher level of concerns about their personal safety due to the political situation in the Middle East? Do they have a lower threshold for pain sensation, or a lower threshold for reporting about pain?

In our study girls reported a higher prevalence of headaches and other somatic complaint when compared to boys in the two ethnic groups (Tables 2, 4, 5). These findings are in accordance with previously published studies: Ostberg et al. investigated headache, abdominal pain and difficulty in falling asleep in Swedish schoolchildren aged 10–18 years. They found that girls reported headache and recurrent abdominal pain twice as much as boys, and that economic stress but not social class was a significant determinant of somatic symptoms in Swedish children [22] Kelly et al. investigated psychosomatic symptoms among school children 11–17 years of age in Ireland. Of the somatic symptoms, headache was the most common among both boys and girls, with a steep increase in reports of headaches among girls with age. The proportion of girls reporting weekly headaches and abdominal pain at 17 years of age was (44.4% and 23.8%, respectively. This prevalence was approximately twice that of boys of the same age (23.1% and 10.2%) [23].

In our study we also found that other somatic complaints such as abdominal pain, palpitations, disordered sleep and fatigue were more frequent in students who suffered from headaches, and that it was held true for both ethnic groups.

These findings are also in accordance with the study published by Ghandour et al. [1] who reported high prevalence of combination of abdominal pain (40.8%) and headache (30%), among tenth grade American girls.

### Study limitations

Our study has a few limitations. Since the study was done in high schools in northern Israel, it does not necessarily represent the general population of the country. We did not look into socioeconomic differences between the two ethnic groups as well as risk factors such as coffee drinking, cigarette smoking, and stress levels that could have an impact on headache prevalence.

## Conclusions

Frequent headaches of at least one headache episode per week are a common complaint among Israeli high-school students (22.6% of all participants), more in girls than in boys, more among the Jewish students than among their Arab peers. Once a student complains of headaches, it is more likely that he or she would have another somatic complaint, mostly abdominal pain or disordered sleep. There is a significant difference between the two ethnic groups in regards to the prevalence of headaches and other somatic complaints (higher prevalence in Jews), a difference that warrants further investigation to be fully explained. It is important for any health care professional that provides consultation and/ or treatment for adolescents to become aware of ethnic and gender differences in regards to health complaints in his/ her catchment area so to better provide a tailored approach to his/her adolescent patients.

## Competing interest

None of the authors has any conflict of interest to disclose.

## Authors’ contribution

NC conceives of the study, and participated in its design and coordination and helped to draft the manuscript. JG participated in the design of the study and wrote the first draft. IC participated in the design of the study and helped to draft the manuscript. All authors read and approved the final manuscript.

## References

[B1] Ghandour RM Overpeck MD Huang ZJ Kogan MD Scheidt PC Headache, stomachache, backache, and morning fatigue among adolescent girls in the United States: associations with behavioral, sociodemographic, and environmental factors Arch Pediatr Adolesc Med 2004 14 8 797 803 10.1001/archpedi.158.8.797 15289254

[B2] Belmaker E Non-specific somatic symptoms in early adolescent girls J Adolesc Health Care 1984 14 30 33 10.1016/S0197-0070(84)80242-9 6607248

[B3] Larsson BS Somatic complaints and their relationship to depressive symptoms in Swedish adolescents J Child Psychol Psychiatry 1991 14 821 832 10.1111/j.1469-7610.1991.tb01905.x 1918231

[B4] Egger HL Costello EJ Erkanli A Angold A Somatic complaints and psychopathology in children and adolescents: stomachaches, musculoskeletal pains and headaches J Am Acad Child Adolesc Psychiatry 1999 14 852 860 10.1097/00004583-199907000-00015 10405503

[B5] Kristja’nsdo’ttir G Prevalence of pain combinations and overall pain: a study of headache, stomach pain and back pain among school-children Scand J Soc Med 1997 14 58 63 10.1177/140349489702500112 9106948

[B6] Howard RF Current status of pain management in children JAMA 2003 14 2464 2469 10.1001/jama.290.18.2464 14612483

[B7] Rhee H Prevalence and predictors of headaches in US adolescents Headache 2000 14 528 538 10.1046/j.1526-4610.2000.00084.x 10940091

[B8] King S Chambers CT Huguet A The epidemiology of chronic pain in children and adolescents revisited: A systematic review Pain 2011 14 12 2729 38 10.1016/j.pain.2011.07.016 22078064

[B9] Abu-Arafeh I Razak S Sivaraman B Graham C Prevalence of headache and migraine in children and adolescents: a systematic review of population-based studies Dev Med Child Neurol 2010 14 12 1088 1097 10.1111/j.1469-8749.2010.03793.x 20875042

[B10] Nikiforow R Hokkanen E An epidemiological study of headache in an urban and a rural population in northern Finland Headache 1978 14 3 137 145 10.1111/j.1526-4610.1978.hed1803137.x 669944

[B11] Rho YI Chung HJ Lee KH Prevalence and clinical characteristics of primary headaches among school children in South Korea: A nationwide survey Headache 2012 14 4 592 599 10.1111/j.1526-4610.2011.02001.x 21929660

[B12] Chong SC Chan YH Ong HT Low PS Tay SK Headache diagnosis, disability and co-morbidities in a multi-ethnic, heterogeneous paediatric Asian population Cephalalgia 2010 14 8 953 961 20656706 10.1177/0333102409356327

[B13] Lucchetti G Peres MF The prevalence of migraine and probable migraine in a Brazilian favela: results of a community survey Headache 2011 14 6 971 979 10.1111/j.1526-4610.2011.01899.x 21631479

[B14] Rhee H Additional thoughts about racial differences in the prevalence of headaches in US adolescents Headache 2001 14 4 419 420 10.1046/j.1526-4610.2001.111006419.x 11318892

[B15] Bullinger M Anderson R Cella D Aaronson N Developing and evaluating cross-cultural instruments from minimum requirements to optimal models Quality of Life Research 1993 14 451 459 10.1007/BF00422219 8161979

[B16] Karli N Akis N Zarifoğlu M Headache prevalence in adolescents aged 12 to 17: a student-based epidemiological study in Bursa Headache 2006 14 4 649 655 10.1111/j.1526-4610.2006.00362.x 16643560

[B17] Stewart W Lipton R Celentano D Reed M Prevalence of migraine headache in the United tates. Relation to age, income, race, and ther sociodemographic factors JAMA 1992 14 64 69 10.1001/jama.1992.03480010072027 1727198

[B18] Stang P Osterhaus J Impact of migraine in the United States: Data from the National Health Interview Survey Headache 1993 14 29 35 10.1111/j.1526-4610.1993.hed3301029.x 8436495

[B19] Al Jumah M Awada A Al Azzam S Headache syndromes amongst schoolchildren in Riyadh, Saudi Arabia Headache 2002 14 281 286 10.1046/j.1526-4610.2002.02081.x 12010385

[B20] Isik U Ersu RH Ay P Prevalence of headache and its association with sleep disorders in children Pediatr Neurol 2007 14 146 151 10.1016/j.pediatrneurol.2006.11.006 17352946

[B21] The Israel Ministry of Industry, Trade and Labor, Arab Academics 2001–2008 from: http://www.moital.gov.il

[B22] Ostberg V Alfven G Hjern A Living conditions and psychosomatic complaints in Swedish schoolchildren Acta Paediatr 2006 14 8 929 934 10.1080/08035250600636545 16882564

[B23] Kelly C Molcho M Doyle P Gabhainn SN Psychosomatic symptoms among schoolchildren Int J Adolesc Med Health 2010 14 2 229 235 21061923 10.1515/ijamh.2010.22.2.229

